# Incidence and clinical characterization of retinal detachment in acute retinal necrosis: a global federated database analysis

**DOI:** 10.3389/fopht.2026.1829400

**Published:** 2026-06-08

**Authors:** Donovin Thompson, Sidra Zafar, Meghan Berkenstock

**Affiliations:** 1 Drexel University College of Medicine, Philadelphia, PA, United States; 2 Wills Eye Hospital, Sidney Kimmel School of Medicine, Philadelphia, PA, United States; 3 Division of Ocular Immunology, Wilmer Eye Institute, Johns Hopkins School of Medicine, Baltimore, MD, United States

**Keywords:** acute retinal necrosis, retinal detachment, retrospective cohort, risk factors, uveitis

## Abstract

**Background:**

To determine the incidence of retinal detachment (RD) following acute retinal necrosis (ARN) and to evaluate demographic and treatment-related associations using a large, multi-institutional database. Design: Retrospective cohort study.

**Methods:**

Setting: TriNetX Research Network. Study population: Patients diagnosed with ARN between January 1, 2014, and January 1, 2024. Procedures: ARN and RD were identified using ICD and CPT codes. Chi-square tests, risk ratio analyses, and logistic regression models were used to evaluate demographic and treatment-related associations of RD. Main outcome measures: Incidence and associations for RD at 3, 6, and 12 months post-diagnosis of ARN.

**Results:**

Among 1,785 patients with ARN, the overall incidence of RD was 12.49% (n=223), with 135 cases (60.5%) occurring within the first 3 months of diagnosis and 88 cases (39.5%) occurring thereafter. Although aggressive combination therapy consisting of systemic corticosteroids, oral valacyclovir, and/or intravitreal foscarnet was primarily used in severe cases, RD still occurred frequently. No significant differences were observed in RD incidence based on age, sex, diabetes status, or immunosuppression.

**Conclusions:**

In this large EHR-based cohort, RD following ARN occurred most commonly within the first 3 months after diagnosis. Treatment exposures (corticosteroids, valacyclovir, intravitreal foscarnet) should be interpreted strictly as proxies of disease severity, not independent risk factors for RD. These findings underscore the importance of close ophthalmic monitoring during the first 3 months after ARN diagnosis, when the risk of RD is highest.

## Background

Acute retinal necrosis (ARN) is a rare but visually devastating condition characterized by peripheral retinal necrosis, occlusive vasculitis, and vitritis (1). The reported annual incidence is approximately 0.63 cases per million persons (2), and it can affect individuals across a wide range of ages and immune statuses (1). Despite prompt antiviral therapy, visual outcomes are often limited by complications such as retinal detachment (RD), optic neuropathy, hypotony, and macular edema (3). Prior studies have estimated that RD occurs in approximately 2% of patients at presentation, increasing to nearly 47% over the disease course (4).

Most existing data on ARN are derived from small case series or single- and multi-center studies, often including fewer than 100 patients (1, 3, 5–10). While these studies have identified potential associations between RD and factors such as viral load, initial visual acuity, and extent of retinal necrosis (5, 11), the limited sample sizes and heterogeneity of study populations restrict generalizability. Additionally, few large-scale analyses have examined RD outcomes in relation to demographic factors, comorbid conditions, and real-world treatment patterns (12, 13).

Prior work has also demonstrated variation in viral etiologies across demographic groups, with varicella zoster virus more commonly observed in older and immunocompromised patients and herpes simplex virus more frequently identified in younger individuals (11). However, these differences have not consistently translated into differences in RD incidence (11). Similarly, studies evaluating treatment strategies have reported mixed findings, with some suggesting benefit from combined systemic antiviral therapy and early surgical intervention (4), while others have found no significant differences in RD rates across antiviral regimens (8, 13).

Given these limitations, there remains an incomplete understanding of RD incidence and associated clinical factors in ARN at a population level. The present study utilizes a large, multi-institutional electronic health record database to characterize RD incidence and examine demographic and treatment-related associations in a real-world cohort of patients with ARN.

## Methods

### Study design and data source

This retrospective cohort study utilized the TriNetX Global Collaborative Network, a federated database of de-identified electronic health records from 163 healthcare institutions. The study was approved by the Drexel University College of Medicine Institutional Review Board and adhered to the tenets of the Declaration of Helsinki.

### Study population

Patients diagnosed with acute retinal necrosis between January 1, 2014, and January 1, 2024, were identified using ICD-10 codes (H30.13, H30.131, H30.132, H30.133, H30.139). Patients with a history of retinal detachment repair prior to ARN diagnosis were excluded. All analyses were performed at the patient level (rather than the eye level), as laterality-specific ophthalmic data are inconsistently available within TriNetX.

### Outcomes

The primary outcome was the incidence of RD following ARN diagnosis. RD was identified using ICD codes (H33.00–H33.05, H33.4) and CPT codes for RD repair (67105, 67107, 67108, 67113). Incidence was calculated overall and at 3, 6, and 12 months following diagnosis.

### Treatment variables

Treatment patterns were assessed within 365 days of ARN diagnosis and included oral valacyclovir, oral and intravenous acyclovir, intravitreal foscarnet, intravenous dexamethasone, and oral prednisone. Corticosteroid implant use (fluocinolone or dexamethasone) within 6 months prior to diagnosis was also evaluated.

### Covariates

Demographic variables collected included age, sex, race, and ethnicity. Race was categorized as White versus Non-White (including Black, Asian, Native American, and Other) for analyses due to small cohort sizes, limiting subgroup analysis of individual races. Comorbid conditions including diabetes mellitus, HIV, and cancer were also collected.

Propensity score matching was performed using age, sex, race, and ethnicity to reduce baseline differences between cohorts, with standardized mean differences less than 0.1 indicating adequate balance.

### Statistical analysis

Incidence rates were calculated at predefined time intervals. Group comparisons were performed using chi-square tests for categorical variables and independent sample t-tests for continuous variables. Logistic regression models were used to evaluate associations between corticosteroid exposure and RD. Risk ratios and risk differences were calculated for selected treatment and comorbidity comparisons using the TriNetX platform.

## Results

From 179,121,826 patients in the database, 1,785 (0.001%) were identified with a diagnosis of ARN. The cohort was predominantly male (52.66%), White (63.64%), and non-Hispanic (72.16%), with a mean age of 60 years (SD 19; range 2–90) (**Table 1**). When race was dichotomized, no significant difference was observed between patients who developed RD and those who did not (OR 0.82, 95% CI [0.61–1.11], p = 0.208). No significant differences were observed in age, sex, or ethnicity. Among comorbid conditions, no differences in RD incidence were observed based on diabetes mellitus, HIV status, or cancer status **Table 2**.

**Table 1:** Baseline demographic and clinical characteristics of patients with acute retinal necrosis (ARN), stratified by retinal detachment (RD) status.

Characteristic	All ARN patients (N = 1,785)	Retinal detachment (n = 223)	No retinal detachment (n = 1,562)
Age (years)			
Mean ± SD	60 ± 19	59 ± 19	60 ± 19
Range	2–90	5–90	2–90
Sex, n (column %)			
Male	940/1,785 (52.7%)	129/223 (57.8%)	811/1,562 (51.9%)
Female	845/1,785 (47.3%)	94/223 (42.2%)	751/1,562 (48.1%)
Ethnicity, n (column %)			
Not Hispanic or Latino	1,288/1,785 (72.2%)	178/223 (79.8%)	1,110/1,562 (71.1%)
Hispanic or Latino	96/1,785 (5.4%)	15/223 (6.7%)	81/1,562 (5.2%)
Unknown	401/1,785 (22.5%)	30/223 (13.5%)	371/1,562 (23.8%)
Race, n (column %)			
White	1,136/1,785 (63.6%)	142/223 (63.7%)	994/1,562 (63.6%)
Black or African American	316/1,785 (17.7%)	40/223 (17.9%)	276/1,562 (17.7%)
Asian	63/1,785 (3.5%)	10/223 (4.5%)	48/1,562 (3.1%)
American Indian or Alaska Native	12/1,785 (0.7%)	10/223 (4.5%)	11/1,562 (0.7%)
Native Hawaiian or Other Pacific Islander	10/1,785 (0.6%)	0/223 (0.0%)	10/1,562 (0.6%)
Other	96/1,785 (5.4%)	15/223 (6.7%)	86/1,562 (5.5%)
Unknown	159/1,785 (8.9%)	15/223 (6.7%)	144/1,562 (9.2%)
Comorbidities, n (column % of full cohort)			
HIV (without cancer)	112/1,785 (6.3%)	<10	
Cancer (without HIV)	398/1,785 (22.3%)	<10	
Type 1 Diabetes Mellitus	71/1,785 (4.0%)	13/223 (6.0%)	62/1,562 (4.0%)
Type 2 Diabetes Mellitus	393/1,785 (22.0%)	58/223 (26.0%)	328/1,562 (21.0%)

ARN, acute retinal necrosis; RD, retinal detachment; SD, standard deviation; HIV, human immunodeficiency virus; DM, diabetes mellitus. Percentages are column percentages, representing the proportion of patients within each column (All ARN, RD, or No RD) who belong to a given category. Cells with fewer than 10 patients are suppressed per TriNetX data privacy policy and reported as <10.

**Table 2:** Treatment patterns among patients with acute retinal necrosis (ARN), stratified by retinal detachment (RD) status.

Treatment	All ARN patients (N = 1,785)	Retinal detachment (n = 223)	No retinal detachment (n = 1,562)
Corticosteroids, n (column %)			
Any glucocorticoid	999/1,785 (56.0%)	185/223 (83.0%)	814/1,562 (52.1%)
Oral prednisone (0–12 months)	395/1,785 (22.1%)	79/223 (35.4%)	316/1,562 (20.2%)
Oral dexamethasone (0–12 months)	53/1,785 (3.0%)	<10	44/1,562 (2.8%)
Dexamethasone implant (0–12 months)	59/1,785 (3.3%)	<10	53/1,562 (3.9%)
Injectable dexamethasone (0–12 months)	191/1,785 (10.7%)	73/223 (32.7%)	118/1,562 (7.6%)
Antivirals, n (column %)			
Oral valacyclovir (0–3 months)	353/1,785 (19.8%)	115/223 (51.6%)	238/1,562 (15.2%)
Oral valacyclovir (0–12 months)	429/1,785 (24.0%)	131/223 (58.7%)	298/1,562 (19.1%)
Oral acyclovir (0–12 months)	88/1,785 (4.9%)	21/223 (9.4%)	67/1,562 (4.3%)
Intravenous acyclovir (0–12 months)	124/1,785 (6.9%)	37/223 (16.6%)	87/1,562 (5.6%)
Intravitreal foscarnet (0–3 months)	136/1,785 (7.6%)	54/223 (24.2%)	82/1,562 (5.3%)
Intravitreal foscarnet (0–12 months)	140/1,785 (7.8%)	55/223 (24.7%)	85/1,562 (5.4%)
Other, n (column %)			
Prophylactic laser retinopexy	34/1,785 (1.9%)	12/223 (5.4%)	22/1,562 (1.4%)

ARN, acute retinal necrosis; RD, retinal detachment; IV, intravenous; IVT, intravitreal. Percentages are column percentages, representing the proportion of patients within each column (All ARN, RD, or No RD) who received a given treatment. Cells with fewer than 10 patients are suppressed per TriNetX data privacy policy and reported as <10. Treatments were assessed within the specified time window following ARN diagnosis. Injectable dexamethasone reflects systemic (intravenous) administration; intravitreal dexamethasone implant is reported separately.

### Retinal detachment following ARN

The overall incidence of RD following ARN diagnosis was 12.49% (n=223). Of these, 135 cases (60.5%) occurred within the first 3 months of diagnosis, with the remaining 88 cases occurring after 3 months. When stratified by time period, RD incidence did not differ significantly between patients diagnosed from 2014–2018 and those from 2018-2024 (p = 0.75). This stratification was performed to examine whether changes in clinical practice, diagnostic awareness, or treatment approaches over time may have influenced RD rates.

### Treatment patterns

#### Antiviral medications

Within one year of ARN diagnosis, 24% of patients received oral valacyclovir, 7.84% received intravitreal foscarnet, 4.92% received oral acyclovir, and 6.95% received intravenous acyclovir.

#### Corticosteroid medications

Overall, 55.97% of patients received corticosteroids. Oral prednisone (22.13%) and intravenous dexamethasone (10.7%) were the most frequently used agents. Corticosteroid use was more frequently observed among patients who developed RD. In logistic regression models, both intravenous dexamethasone and oral prednisone use were significantly associated with RD.

#### Retinal detachment associations

Within 3 months of ARN diagnosis, RD incidence was higher among patients who received corticosteroids compared to those who did not (RR 1.47, 95% CI [1.002–2.155], p = 0.038). Similarly, higher RD incidence was observed among patients receiving oral valacyclovir and intravitreal foscarnet compared to those not receiving these treatments. No significant differences in RD incidence were observed between intravenous and oral acyclovir or between patients with and without immunosuppression.

Among patients diagnosed with ARN, 4.09% had received corticosteroid implants within 6 months prior to diagnosis. Due to data suppression for small cohort sizes, further subgroup analysis could not be performed.

## Discussion

In this analysis of an international database, we found the overall rate of RD development following a diagnosis of ARN was 12.49% (n=223), with 135 cases (60.5%) occurring within the first 3 months of diagnosis. Prior studies on ARN, most of which were single-center case series, have reported RD rates ranging from 30–75% (6). In a recent IRIS registry analysis of eyes with suspected ARN, the reported RD rate was 20% (13). Differences in treatment patterns provide important context for this discrepancy. In the IRIS cohort, nearly 28% of patients received adjunctive therapies such as intravitreal antivirals or early pars plana vitrectomy, which are typically reserved for more severe disease. In contrast, utilization of intravitreal and intravenous therapies in our cohort was lower, which may reflect inclusion of patients with earlier or less severe presentations and contribute to the lower RD incidence observed. However, because detailed clinical variables such as extent of retinal necrosis and degree of inflammation are not available within TriNetX, baseline disease severity cannot be directly assessed. Importantly, the findings of this study should be interpreted as descriptive associations within an EHR-based dataset rather than evidence of independent predictors of RD.

Standard management of ARN involves an induction phase of high-dose systemic antiviral therapy, either intravenous acyclovir (10–13 mg/kg every 8 hours) or oral valacyclovir (2 g three times daily) for 7–14 days, often combined with intravitreal foscarnet (1.2–2.4 mg/0.1 mL twice weekly) in cases of severe or rapidly progressive disease (14). This is followed by oral antiviral maintenance therapy, typically valacyclovir 1 g daily, continued for 6 weeks to several months depending on clinical response. In our cohort, 24.0% of patients received oral valacyclovir and 7.8% received intravitreal foscarnet within 12 months of diagnosis, while 6.9% received intravenous acyclovir. A subset of patients had no documented antiviral therapy, which likely reflects incomplete medication capture within TriNetX rather than true absence of treatment, as antivirals are considered standard of care for ARN. Notably, the majority of RD events in our cohort occurred within the first 3 months of diagnosis (135 of 223 cases, 60.5%). Although direct stratification of RD events by antiviral route could not be performed, this temporal pattern underscores the importance of close ophthalmic monitoring during this early period of highest risk.

When analyzing demographics such as age, prior studies have reported conflicting results regarding its association with RD risk following ARN. Some studies suggest increased risk in younger patients (15), potentially due to a more robust inflammatory response, whereas others report better visual outcomes in younger individuals (3). Conversely, delayed presentation and age-related ocular changes have been proposed as contributors to worse outcomes in older patients (16, 17). In our cohort, age was not significantly associated with RD development; however, these findings should be interpreted cautiously given the absence of detailed clinical data.

Similarly, diabetes mellitus was not significantly associated with RD development. While immunosuppressed individuals are more likely to develop ARN (10), prior studies have not demonstrated an increased risk of RD in this population (11). Our findings were consistent, with no significant difference in RD incidence between patients with and without cancer or HIV. However, these groups are heterogeneous, and the lack of laboratory data and treatment information limits assessment of the true degree of immunosuppression.

Although race was explored as a potential factor associated with RD following ARN, no statistically significant association was identified (OR 0.82, 95% CI [0.61–1.11], p = 0.208). Prior ARN literature has similarly been limited in evaluating racial differences in outcomes; Bevinger et al. reported no significant racial differences in viral etiology, though RD risk by race was not specifically assessed. More broadly, studies in viral retinitis by Zhou et al. have demonstrated that socioeconomic disadvantage may be associated with worse visual outcomes, increased missed appointments, and higher complication rates, suggesting that structural and healthcare access factors may still influence disease course (18). In ARN, where delays in diagnosis and antiviral treatment may worsen prognosis, these factors remain clinically relevant. However, important clinical variables such as visual acuity at presentation, extent of retinal necrosis, timing of treatment initiation, and Standardization of Uveitis Nomenclature (SUN) criteria are not available within TriNetX, limiting interpretation of demographic associations. While race was not significantly associated with RD development in this cohort, the role of socioeconomic and healthcare access factors in ARN outcomes remains incompletely understood and warrants further investigation.

Systemic corticosteroid use is typically reserved for patients with more severe inflammation (19, 20). In our cohort, corticosteroid exposure was more common among patients who developed RD, and analysis demonstrated an association with early RD RR 1.47, 95% CI [1.002–2.155], p = 0.038). However, this finding should be interpreted cautiously, as it likely reflects confounding by indication, with corticosteroids used in patients at higher baseline risk. Additionally, the temporal relationship between corticosteroid use and RD diagnosis cannot be precisely determined. Similarly, oral valacyclovir and intravitreal foscarnet use within the first 3 months after ARN diagnosis were associated with higher RD rates. These associations likely reflect treatment selection in more severe cases rather than causal effects, as patients requiring aggressive antiviral therapy often have more extensive or rapidly progressive disease.

A key strength of this study is the use of the TriNetX database, which aggregates data from over 170 million patients across diverse healthcare settings, allowing for a large-scale assessment of a rare condition. The scientific value of this work lies primarily in its ability to characterize the real-world epidemiology of ARN-associated RD across a diverse, multi-institutional population at a scale not achievable with single-center studies. By establishing population-level incidence estimates, identifying the critical early risk window within 3 months of diagnosis, and demonstrating that common demographic and comorbidity variables are not independently associated with RD in this dataset, this study provides a descriptive foundation that can inform prospective study design, patient counseling, and clinical monitoring protocols. However, several limitations must be considered. Because identification relied on diagnostic coding, confirmation of ARN using polymerase chain reaction testing or standardized clinical criteria such as the SUN criteria was not available; included cases may therefore represent clinically diagnosed or suspected ARN rather than laboratory-confirmed disease. Additionally, the first recorded ARN diagnosis code may not correspond to the true onset of disease, introducing potential ambiguity in index dates and timing of subsequent events. Because analyses were performed at the patient level, eye-specific outcomes and bilateral disease involvement could not be assessed. TriNetX does not distinguish between intravenous and intravitreal routes for certain medications, categorizing them broadly as injectable, which introduces potential misclassification and limits interpretation of treatment patterns. Important clinical variables, including extent of retinal necrosis, degree of inflammation, viral etiology, baseline disease severity, and visual acuity at presentation, are also unavailable. Patients with more severe disease may undergo more frequent follow-up evaluations, increasing the likelihood of RD detection and introducing potential surveillance bias. Medication data may not fully capture prescribing accuracy, treatment initiation, adherence, or completion. Similarly, coding for prophylactic laser procedures or vitreoretinal surgery may not reliably distinguish preventive from therapeutic interventions. RD events occurring outside participating systems, patient transfer, loss to follow-up, or death may also not be captured, potentially leading to underestimation of incidence. Given the limitations inherent to retrospective EHR-based data, findings should be interpreted as associative rather than causal.

Despite these limitations, this study provides a large-scale, real-world descriptive overview of ARN and RD outcomes. The observed RD incidence was lower than that reported in prior studies, likely reflecting differences in case capture and baseline disease severity. Across available variables, age, sex, diabetes, and immunosuppression were not associated with RD development within the constraints of this dataset. Treatment exposures, including corticosteroids, valacyclovir, and intravitreal foscarnet, should be interpreted strictly as proxies of disease severity, not as independent predictors of RD outcome. Clinically, these findings emphasize the importance of early recognition and close monitoring of patients with ARN, particularly within the first three months after diagnosis, when the majority of RD events occur.

## Data Availability

The original contributions presented in the study are included in the article/supplementary material. Further inquiries can be directed to the corresponding author.
